# Fairness in Mobile Phone–Based Mental Health Assessment Algorithms: Exploratory Study

**DOI:** 10.2196/34366

**Published:** 2022-06-14

**Authors:** Jinkyung Park, Ramanathan Arunachalam, Vincent Silenzio, Vivek K Singh

**Affiliations:** 1 School of Communication & Information Rutgers University New Brunswick, NJ United States; 2 Department of Computer Science Rutgers University New Brunswick, NJ United States; 3 School of Public Health Rutgers University Newark, NJ United States; 4 Institute for Data, Systems, and Society Massachusetts Institute of Technology Cambridge, MA United States

**Keywords:** algorithmic bias, mental health, health equity, medical informatics, health information systems, gender bias, mobile phone

## Abstract

**Background:**

Approximately 1 in 5 American adults experience mental illness every year. Thus, mobile phone–based mental health prediction apps that use phone data and artificial intelligence techniques for mental health assessment have become increasingly important and are being rapidly developed. At the same time, multiple artificial intelligence–related technologies (eg, face recognition and search results) have recently been reported to be biased regarding age, gender, and race. This study moves this discussion to a new domain: phone-based mental health assessment algorithms. It is important to ensure that such algorithms do not contribute to gender disparities through biased predictions across gender groups.

**Objective:**

This research aimed to analyze the susceptibility of multiple commonly used machine learning approaches for gender bias in mobile mental health assessment and explore the use of an algorithmic disparate impact remover (DIR) approach to reduce bias levels while maintaining high accuracy.

**Methods:**

First, we performed preprocessing and model training using the data set (N=55) obtained from a previous study. Accuracy levels and differences in accuracy across genders were computed using 5 different machine learning models. We selected the random forest model, which yielded the highest accuracy, for a more detailed audit and computed multiple metrics that are commonly used for fairness in the machine learning literature. Finally, we applied the DIR approach to reduce bias in the mental health assessment algorithm.

**Results:**

The highest observed accuracy for the mental health assessment was 78.57%. Although this accuracy level raises optimism, the audit based on gender revealed that the performance of the algorithm was statistically significantly different between the male and female groups (eg, difference in accuracy across genders was 15.85%; *P*<.001). Similar trends were obtained for other fairness metrics. This disparity in performance was found to reduce significantly after the application of the DIR approach by adapting the data used for modeling (eg, the difference in accuracy across genders was 1.66%, and the reduction is statistically significant with *P*<.001).

**Conclusions:**

This study grounds the need for algorithmic auditing in phone-based mental health assessment algorithms and the use of gender as a protected attribute to study fairness in such settings. Such audits and remedial steps are the building blocks for the widespread adoption of fair and accurate mental health assessment algorithms in the future.

## Introduction

### Background

Various machine learning (ML) algorithms are increasingly being used to make crucial decisions previously made by humans. Whether they are involved in approving loans, granting college admissions, or identifying the need for additional health support, automated algorithms find patterns, predict outcomes, and make decisions that may have consequential impacts on individuals’ lives [1]. Indeed, the dependency on algorithms has eased our lives by replacing subjective human decisions with ML algorithms. The movement toward the application of automated algorithms in the health domain was not an exception. For instance, the proactive assessment of an individual’s mental health is essential for maintaining a healthy and well-functioning society [2]. Although this holds the promise of dramatically wider access to mental health care, it is also fraught with inequities that can often inadvertently be baked into the algorithmic prediction of mental health levels.

ML algorithms attempt to find the generalized pattern from the training data, and sometimes these algorithms can manifest inherent biases across demographic characteristics such as age, race, ethnicity, and gender. A reason for the existing biases can be explained by *negative legacy* [3] (ie, the absence of sufficient data for a particular demographic group). For example, giving loans mostly to higher-income groups in the past may result in disapproval of loans to lower-income groups by algorithms that were informed by historical data, resulting in potential damage to individuals belonging to lower-income groups.

Such biases can be especially deleterious if they are part of health care algorithms. For instance, a recent study by Allen et al [4] found that algorithms used to assess mortality scores exhibit differential accuracy across races, thereby increasing racial disparities in health care. Similarly, Gianfrancesco et al [5] demonstrated that algorithmic predictions based on electronic health records can discriminate against multiple demographic groups. In particular, Obermeyer et al [1] showed that existing algorithms do not adequately identify the need for health support for people of color.

Building on these trends, we move the discussion of algorithmic fairness to mobile mental health assessment algorithms, which have been increasingly used in recent times [6]. With >6 billion users, mobile phones are one of the most ubiquitous consumer devices in the world. Many of them (especially smartphones) have capabilities conducive to monitoring an individual’s physical activity, location, and communication patterns, each of which has been connected to mental health in the past [7,8]. Thus, mobile phones hold significant promise as a platform for monitoring multiple indicators of mental health risks and improving long-term management and treatment delivery to people with mental health issues [7,9]. At the same time, the creation of phone data–based ML models without considering the aspects of justice and fairness could reify, amplify, and multiply existing health disparities for certain segments of society (eg, women). Considering the abovementioned factors, the main research questions (RQs) of this study were as follows:

- RQ1: Are mobile phone-based mental health algorithms susceptible to bias in terms of gender?
- RQ2: Is it possible to reduce the level of bias while maintaining high accuracy?

### Related Work

#### Predicting Mental Health

Over the past few decades, mental health has typically been assessed based on self-reported surveys that involved sporadic sampling, most of which were initiated after some significant events had taken place in an individual’s life. Recently, as the availability of mobile phone data has increased, several studies have suggested using mobile phone data to detect and predict mental health conditions. Wang et al [10] introduced a mobile phone sensing system to automatically infer mental well-being, including depression, stress, flourishing, and loneliness. The study reported that automatically sensed conversation, activity, mobility, and sleep were significantly associated with mental health outcomes. By collecting data from sensors in mobile phone users (eg, location, messages, and calls), a longitudinal study showed a relationship between users’ routines and moods [11]. Another study also found that mobile phone–based features such as call count, call response rate, and the number of new contacts are positively associated with mental health [8]. Using location information collected by a mobile phone app, Canzian and Musolesi [12] assessed the correlation between mobility patterns and the presence of depressive mood. A similar study also presented the relationship between depressive symptoms and the use of mobile phones and the movement through geographic spaces [7].

The results of the abovementioned studies provide clear evidence of interconnections between mobile phone data features and mental health conditions. More importantly, they suggested the potential of developing phone-based ML algorithms as a basis for the unobtrusive prediction of mental health conditions. However, to the best of our knowledge, no study has examined the possibility of algorithmic bias in predicting mental health status by using mobile phone data. Motivated by previous work on algorithmic fairness (see the *Algorithmic Fairness* section), this study attempted to mitigate the discriminatory impact of gender on mental health prediction algorithms.

#### Algorithmic Fairness

An increasing amount of research has suggested that ML algorithms in many domains are not free from discriminatory decision-making. Even with the best intentions, data-driven algorithmic decision-making processes can reproduce, inherit, or reflect the existing social biases. Algorithmic bias may stem from different sources, including (1) input data that may have unequal representation from different groups, (2) an algorithm that has been inadvertently or knowingly coded to make unfair decisions, (3) misuse of certain models in a different context, and (4) biased training data, which reaffirms that social biases may be used as evidence that an algorithm performs well [13]. Broadly, the sociotechnical system framework underscores that the value system of the algorithm developers is coded during the algorithm design process; hence, each assumption (often implicit) made by the developers influences the real-world performance of the algorithm [14].

At the same time, multiple bias mitigation techniques have been developed for fairness in the ML literature [15,16]. Roughly, they attempt to counter such algorithmic bias by modifying the training data (preprocessing), learning algorithms (in-processing), or prediction (postprocessing). Preprocessing approaches focus on adapting the data going into the algorithms [16], in-processing approaches change the core algorithm (eg, change optimization function) [15], and postprocessing algorithms tend to modify the predicted labels to increase fairness [17].

Despite the plethora of related work, attempts to ensure algorithmic fairness toward a protected attribute (gender in our case) in the algorithmic assessment of mental health (high or low) have not been made.

#### Gender Bias

Various attempts have been made to tackle the issue of gender bias in computer algorithms by auditing algorithms for gender bias and modifying algorithms to eliminate stereotypes. For example, a study found that image search results for occupations could amplify gender stereotypes by portraying the minority gender as less professional [18]. Another study found gender stereotypes in word embeddings (eg, a framework to represent text data as vectors) and created debiasing algorithms to reduce gender bias while preserving the utility of the embeddings [19]. Furthermore, Zhao et al [20] tackled the problem of the effect of data imbalance, arguing that such data imbalance can worsen discrimination in terms of gender. They quantified the biases in visual recognition models and calibrated the models to reduce bias. However, no research has been conducted on gender equality using classification algorithms that predict mental health.

This study addressed the problem of identifying and reducing gender bias, as the overrepresentation of men in training data could accelerate gender inequality in mental health prediction algorithms. Particularly, we focused on the issue of *negative* *legacy*, as suggested by Kamishima et al [3], which involves the idea that unfair sampling or labeling in the training data may lead to a disparate impact [16,21] on a certain group of people (eg, granting loans mostly to those who with higher income in the past may result in disapproval of loans to those with low income by the algorithms).

#### Perspective on Fairness and Justice

There exist multiple interpretations of fairness in the algorithmic fairness literature [22]. For instance, scholars define fairness in terms of maximizing utility for groups or respecting various rules such as individual rights and freedoms [23,24]. However, other interpretations abound, some of which are mutually incompatible [25].

The most commonly used approaches are those based on *distributive* and *procedural* justice [22]. While distributive justice focuses on how outcomes are distributed across the population, procedural justice focuses on the processes used to undertake the decisions [26,27].

An influential philosophical theory of fairness is attributed to the 20th-century philosopher Rawls, who equated fairness and justice, arguing broadly that fairness is *a demand for impartiality* [21,22]. In this study, we followed the approach for distributive justice based on the interpretation of Rawls. Specifically, we considered an algorithm to be fair if its performance did not vary for individuals with different demographic descriptors (eg, gender).

This is related to the concept of *disparate impact* [28]. Disparate impact, in US labor law, refers to practices in areas such as employment and housing, which affect one group of people of a protected characteristic more adversely than another, even when the rules applied by employers or landlords appear to be neutral [29]. Most federal civil rights laws protect against disparate impacts based on race, color, religion, national origin, and sex as protected traits, and some laws include disability status and other traits.

## Methods

### Data Set

We used a labeled data set from a previous study by Singh and Long [8], which explored the associations between call log data and mental health based on a 10-week field and laboratory study. The data set included phone-based behavioral data and self-reported mental health survey data. Phone-based data (eg, call volume, interaction dynamics, diversity in contacts, tie strength, and temporal rhythms) were collected through the app installed on each participant’s mobile phone. Meanwhile, mental health was measured via in-person survey sessions using the Mental Health Inventory subscale of the 36-Item Short Form Health Survey [30]. After passing a preprocessing and classification process, the study showed that automated ML algorithms using phone-based features achieved up to 80% accuracy in automatically classifying the mental health level (above or below the mean) of an individual [8].

A total of 59 participants completed the survey administered by Singh and Long [8]. However, some participants did not complete all the surveys, and some did not enter the correct identifier (International Mobile Equipment Identity [IMEI] number) consistently across surveys. This resulted in a subset of 45 participants in the study [8]. For this study, we returned to the survey data and decided to manually handle the *off-by-one* errors (ie, the mismatch in IMEI for different surveys only by 1 digit). Given that IMEI numbers have 14 to 15 digits, in the approximately 60-participant sample size, we considered the odds of 2 participants to be off by just 1 digit without human error being extremely low. This process helped us obtain a complete data set (ie, phone data, a mental health survey, and a demographic survey) for 55 participants.

The data set we obtained from Singh and Long [8] had gender as a demographic attribute that we considered a protected attribute. Note that a protected attribute in the algorithmic fairness literature is one on which performance should not depend [15]. Among these 55 participants, 21 (38%) self-reported their gender as women or female (minority class), and 34 (62%) self-described as men or male. Note that this study does not differentiate between (biological) sex and (socially construed) gender. In addition, note that we consider the use of binary gender as a limitation of this study. Future studies should be conducted, which include participants with nonbinary gender identities.

### Preprocessing and Model Training

The initial obtained data set was imbalanced (ie, there was not enough data for one class), which is a common problem in the fairness literature [31]. To mitigate the effect of imbalance, we applied the synthetic minority oversampling technique [32] to the training data (the test data remained in the original ratio). This technique works in balancing the data set by generating synthetic observations based on the existing minority observations.

Before moving on to the application of any ML algorithm, the missing values were filled with the median values of the corresponding features. To reduce the impact of features with high variance, the features were standardized by removing the mean and scaling to unit variance. To build a classification model for high or low mental health scores, instances were labeled into 2 categories (1=high and 0=low) via a median split.

With small sample data and high-dimensional space, there is always a chance of overfitting and reduced generalization. To avoid these issues, we used principal component analysis [33]. Principal component analysis confirmed that the top 5 components explained >99% of the variance (the larger the variation across a dimension, the more the information it contains); hence, we used the top 5 components as features for model creation.

The abovementioned latent features were passed to several classification algorithms to classify the level of mental health (ie, whether the score was above or below the mean score of the population). As the sample data size was relatively modest, we refrained from splitting the data set into training and test sets. Instead, as suggested by prior literature [8,34], we applied 5-fold cross-validations and experimented with 5 popular classification algorithms, including logistic regression, support vector machine, random forest, k-nearest neighbors, and multilayer perceptron neural networks using the *scikit-learn* library [35]. We ran all algorithms for 100 iterations, and the results are reported in the form of average overall accuracy, male accuracy (ie, accuracy for male individuals), and female accuracy (see the *Results* section).

Using the abovementioned data, we could, in principle, replicate the approach described by Singh and Long [8]. Although the features used were the same, we must note that the implementation was undertaken de novo with different preprocessing steps.

### Auditing Mental Health Algorithms for Bias

Gender was selected as a protected attribute. Following the previous literature [36,37], men were considered the privileged group, and women were considered the unprivileged group. As there are multiple metrics to characterize accuracy in traditional ML (eg, observed accuracy, precision, recall, and F_1_ score), past literature has discussed the need for multiple metrics to characterize bias in ML [13,31]. In this study, we adopted the five most commonly used metrics [15,16,38]:

1. Delta accuracy captures the difference in the accuracy of samples belonging to privileged and unprivileged groups based on sensitive features (eg, gender and race or ethnicity).
2. Delta true positive rate (∆TPR) focuses on equal opportunity for truly deserving entries in both privileged and unprivileged groups to obtain a positive label (eg, higher mental health label) from the algorithm [13,15].
3. Delta false positive rate (∆FPR) ensures that both the true positive rate and the false positive rate (instances where undeserving candidates are granted positive outcomes) are equal across different groups [15,39].
4. Statistical parity difference (SPD) calculates the difference in the probability of favorable outcomes from the algorithm being obtained by the unprivileged group to that of the privileged group [38].
5. Disparate impact captures the ratio of the probability of favorable outcomes for the unprivileged group to that of the privileged group [16] (see Multimedia Appendix 1 [13,15,16,39-41] for more details on the 5 metrics).

Following the principle of disparate impact, a fair information system is one in which the performance does not vary for individuals with different demographic descriptors (eg, gender); hence, the disparate impact metric should be close to 1.0. However, for practical settings, a model is considered biased if its value is <0.8 [40]. Meanwhile, the values of delta accuracy, ∆TPR, ∆FPR, and SPD should be close to zero in fair systems. Following the previous literature [39,41], we used a 2-tailed *t* test to assess whether there was a significant difference in accuracy, true positive rate, and false positive rate levels observed for the privileged and unprivileged groups.

### Reducing Algorithmic Bias in Mental Health Assessment

Disparate impact remover (DIR) [16] is a preprocessing algorithm that modifies the feature values of the data set and makes the algorithm discrimination aware at the time of training. It does not require any changes in the classification algorithm, nor does it amend or postprocess the results of the classification algorithm, thus making it robust and applicable to different algorithms. The scenario in which DIR is needed to preprocess the data set depends on the metric called *balanced error rate (BER),* defined as follows:

BER = (error rate [S = privileged] – error rate [S = unprivileged]) / 2

In algorithmic fairness, the notion of BER is more important than the notion of traditional accuracy as, in most data sets, the contribution of the underprivileged attribute to the entire data set is lesser than that of the privileged attribute. For example, let us consider a data set with 100 rows, where 90 rows belong to the privileged group and 10 rows belong to the unprivileged group. With this data set, if the algorithm predicts all privileged rows right and unprivileged wrong, the error rate would be 10/100, which is 0.1, whereas the BER would be (0+1)/2, which is 0.5.

An approach discussed in the literature [16,17] is to replace the raw values of the data features with normalized variants that capture how extreme the value for an individual (eg, female) stands out within their own demographic group (eg, other women). In particular, the approach suggested by Feldman et al [16] tackles this issue by allowing the considered classes to have equal probabilities of scoring high values for any of the chosen features. With a toy example, where output is college admissions, input is Scholastic Assessment Test (SAT) scores, and with a binary notion of gender (men and women) for the protected class, this approach gives men and women separate scores based on their ranking within their own genders. For example, a man with an 80th percentile SAT score within the men’s group is considered just as worthy as a woman with an 80th percentile SAT score within the women’s group, irrespective of the actual SAT scores. In this way, the approach supports an equitable admission process across 2 genders. Note that in many practical settings, it is useful to undertake *partial repairs* (eg, move the scores at the same percentile across the privileged and unprivileged groups to be closer to each other rather than being congruent). Finally, the above approach can be extended to multidimensional input features for the algorithm. In the considered domain (phone-based mental health assessments), phone use patterns for men and women are known to differ [42,43]. Hence, using the same thresholds for the features (eg, number of phone calls) of men and women as symptoms of mental health issues could yield erroneous and biased results.

In this study, the DIR algorithm for bias reduction was implemented in Python using the IBM AIF360 library [15]. The algorithm was run 100 times, with each iteration having a shuffled version of the data set. The average results for the accuracy and fairness metrics are presented in the *Results* section.

## Results

### Mental Health Assessment Results

Table 1 shows the accuracy of multiple well-known ML algorithms for men and women (averaged over 100 iterations). The best-performing algorithm was random forest, which yielded 78.57% accuracy. These results are similar but not the same as those described by Singh and Long [8]. In both studies, the random forest algorithm yielded the best performance, and the highest observed accuracy was close to 80%. The random forest model with the highest accuracy had 100 estimators or number of trees in the forest and a maximum depth of 6.

**Table 1:** Results showing the average overall accuracy, accuracy for men, and accuracy for women for various machine learning models in mental health assessment (averaged over 100 iterations).

Machine learning models	Overall accuracy (%), mean (SD)	Male accuracy (%), mean (SD)	Female accuracy (%), mean (SD)	Delta across gender (%), mean (SD)	*P* value of the 2-tailed *t* test on delta
Multilayer perceptron neural networks	59.99 (3.67)	58.68 (8.14)	61.92 (9.24)	12.10 (10.41)	<.001
Support vector machine	63.17 (2.91)	65.98 (6.49)	59.60 (8.37)	12.20 (8.67)	<.001
Logistic regression	58.48 (2.69)	66.59 (5.47)	47.38 (6.75)	19.73 (9.80)	<.001
K-nearest neighbors	61.77 (1.78)	70.43 (3.72)	49.63 (5.89)	20.96 (8.46)	<.001
Random forest	78.57 (1.61)	87.16 (2.73)	71.31 (2.51)	15.85 (0.22)	<.001

### Audit Results

We compared the accuracies of different algorithms for the male and female groups (Table 1). The performance was found to be significantly different for the 2 groups in each of the considered algorithms based on a nonpairwise (2-tailed) *t* test (α=.05; *P*<.001) [41]. This indicates that the commonly used ML algorithms, when used for phone-based mental health assessment, are susceptible to bias.

There, a trade-off is expected between accuracy and fairness (ie, with increased fairness, there is typically a dip in accuracy) [31], the random forest model with the highest observed accuracy was selected as the baseline model for further inspection of fairness.

For random forest, the average absolute delta accuracy was 15.85% (Table 2). The absolute values of ∆TPR and ∆FPR were 0.88% and 33.43%, respectively. The average SPD was 26.1%, and the average disparate impact was 0.682, which were distant from the ideal values of 0 and 1.0, respectively.

**Table 2:** The average score for bias metrics in the random forest–based mental health assessment algorithm (average of 100 iterations).

Bias metrics	Observed score, mean (SD)	Ideal score
Delta accuracy (%)	15.85 (0.22)	0
Delta true positive rate (%)	−0.88 (8.39)	0
Delta false positive rate (%)	33.43 (13.50)	0
Statistical parity difference (%)	26.1 (4.16)	0
Disparate impact	0.682 (0.049)	1.0

For 4 of the 5 considered metrics (ie, except ∆TPR), the fairness scores were far from the ideal scores. In other words, the developed model yielded significantly different outcomes for individuals across genders despite reasonable aggregate performance. More precisely, the model was mostly biased against the unprivileged group (in this case, women), and the disparate impact appeared to be a major issue.

### Bias Reduction Results

We recomputed the abovementioned bias metrics after applying the bias reduction algorithm (DIR), and the results averaged over 100 iterations are reported in Table 3. Furthermore, a comparison of the results before and after applying the bias reduction algorithm is presented in Table 4.

**Table 3:** The average score for bias metrics after applying the disparate impact remover approach (average of 100 iterations).

Bias metrics	Observed score, mean (SD)	Ideal score
Delta accuracy (%)	1.66 (1.56)	0
Delta true positive rate (%)	3.74 (6.74)	0
Delta false positive rate (%)	5.58 (9.88)	0
Statistical parity difference (%)	−2.70 (1.71)	0
Disparate impact	1.09 (0.041)	1.0

**Table 4:** Comparison of delta accuracy, statistical parity difference, and disparate impact before and after applying the postprocessing algorithm.

Bias metrics	Baseline model, mean (SD)	After bias reduction, mean (SD)	Difference	*P* values of 2-tailed *t* test on delta
Delta accuracy (%)	15.85 (0.22)	1.66 (1.56)	14.19	<.001
Delta true positive rate (%)	−0.88 (8.39)	3.74 (6.74)	4.63	<.001
Delta false positive rate (%)	33.43 (13.50)	5.58 (9.88)	27.85	<.001
Statistical parity difference (%)	26.10 (4.16)	−2.70 (1.71)	28.80	<.001
Disparate impact	0.682 (0.049)	1.09 (0.041)	0.408	<.001

To test the significance of these improvements, we conducted a 2-tailed *t* test with α=.05 for each of the bias metrics for the before and after scores. The changes in all metrics were noteworthy (*P*<.001). The bias levels were reduced for 4 of the 5 metrics considered in this study. The only exception was ∆TPR, which was the only metric with a low (<5%) score in the baseline condition. This value remained <5% before and after the bias reduction process.

Note that as we move toward making the algorithm less biased, there is often a trade-off that arises in the form of the reduced overall accuracy of the model [13]. The accuracy levels for men and women were 87.16% and 71.31%, respectively (∆accuracy 15.85%; mean 78.50%), before bias reduction. The accuracy levels changed to 78.49% and 76.83% for men and women, respectively (∆accuracy 1.66%; mean 76.83%), after the bias reduction process. The 1.38% reduction (78.50%-77.12%) in the model accuracy was considered an acceptable loss in accuracy for the abovementioned improvements in fairness.

## Discussion

### Principal Findings

#### RQs of the Study

The first RQ in this work was as follows: are mobile phone–based mental health algorithms susceptible to bias in terms of gender?

As summarized in Table 1, we found statistically significant differences across genders in the performance of phone-based mental health assessment algorithms with an array of common ML algorithms. All of these point to the potential for disparate impact across gender with mental health assessment algorithms.

With respect to the performance of the highest accuracy algorithm (using random forest), we found noticeable differences in the performance of the algorithm across genders via the 5 commonly used bias metrics. As shown in Table 2, there was a difference in terms of all 5 metrics between the male and female groups. In particular, we found that the disparate impact ratio was 0.682 in the initial model. However, this value was much lower than the often recommended (and legally accepted) threshold of 0.8, irrespective of the intent of the designers [29]. Although the in-principle replications of algorithms described in the past literature may yield reasonable accuracy, their deployment will require them to meet the legal and ethical guidelines of disparate impact. In addition, similar fairness issues have been well studied in some other spaces (eg, policing and bank loans [44,45]); they are much less explored in algorithmic mental health assessment. However, they will become important with the increased deployment or adoption of mobile mental health tools.

The results also point to another domain in which women are disadvantaged. As per the US Department of Labor Statistics, women earn 82 cents for every dollar earned by men [46]. Similarly, recent research has reported worse performance for women in face recognition [47], Google Translate [48], and image search results [18]. The awareness of such disparities is an important first step in the creation of countermeasures. Broadly, such results in intersection with growing movements such as *Data Feminism* [49] can support the creation of more equitable algorithms. Specifically, we hope that our findings will shed light on the need to ensure fairness in emerging mental health–related domains.

Finally, there are multiple potential reasons for the reduced performance of women in the considered algorithms. Given that the performance is consistently poorer for all the considered ML algorithms (Table 1), possible explanations may lie in the *negative legacy* and *data set imbalance*. Data imbalance is the lack of data samples from a particular demographic group for algorithms to learn from, and negative legacy refers to the lack of positive examples for algorithms to learn from for the unprivileged group [13,31]. For instance, Buolamwini and Gebru [47] argued that a lack of training samples is a reason for poorer performance for women and people of color. Similar to other areas, and perhaps even more urgently, there is a need for more diverse data samples to create accurate and fair ML models in mental health assessment algorithms.

The second RQ in this study was as follows: is it possible to reduce the level of bias while maintaining high accuracy?

On the basis of the results summarized in Table 4, we found that the DIR approach was effective in reducing the disparity in the performance of phone-based mental health assessment algorithms across genders. As reported in Table 4, there were statistically significant differences in terms of all 5 fairness metrics considered upon the application of the DIR approach.

Past literature has discussed the need for multiple metrics to characterize bias in ML [13,31] and that metrics can be orthogonal to each other [25,44]. A suggested process is for system designers to identify a set of parameters that they consider appropriate for a given task [50]. In this study, we considered disparate impact to be an important criterion, considered in consultation with the scores for other fairness metrics. In the considered scenario, noticeably large reductions in bias levels were observed regarding the 4 metrics, except for ∆TPR, where the scores were <5% before and after bias reduction. Finally, we noted that there was a 1.38% decrease in accuracy upon the application of the bias reduction approach.

Overall, we interpreted the results to imply that it is often possible to create fairer versions of algorithms. However, given the variety of fairness metrics that can be considered and the complexities of practical scenarios, the process of bias reduction is likely to involve a human-in-the-loop process and consideration of the trade-offs in terms of multiple metrics [50]. Hence, rather than identifying a silver bullet solution, there might be opportunities for multiple small modifications that allow fairer versions of the algorithms. Having said that, value-sensitive design needs to be an important part of the future design of similar applications [51], and algorithmic audits need to become an essential step in the process of medical approval of newer (algorithmic) diagnostic tools.

The obtained results have multiple implications for different stakeholders engaged in health information systems.

#### Health Informatics Researchers and Policy Designers

This study moves the conversation with health policy designers beyond the equity of the built environment (eg, access to hospitals and parks) to the equity of data infrastructure, which can profoundly influence the health outcomes for millions of individuals going forward [52]. Although there exist multiple legal and policy guidelines that counter the physical aspects of bias (eg, redlining [53]), there is relatively little work on legal and policy frameworks with digital algorithms that undertake similar roles.

#### Health Care Technology Companies

This study identified a feasible pathway for creating algorithms that balance accuracy and equity in the creation of novel health care applications. Hence, the findings support the creation of equitable versions of just-in-time mobile mental health intervention apps.

#### Health Care Providers

This study allows for more robust detection and flagging of mental health issues in patients. Fairer algorithms will reduce the odds of patients being flagged for interventions incorrectly simply because of demographic characteristics, thus allowing for the better alignment of resources between individual providers and the health care industry at large.

#### The Public

The ultimate goal of this study was to create and promote equity in mental health information technology. The fairness of algorithms is intimately connected with trust and adoption. In fact, recent research suggests that disparate impact diminishes consumer trust, even for advantaged users [40]. A robust fair detection process will allow for the scalable delivery of just-in-time and tailored mental health support services to a wider population. This is important, given the huge disparity between the need for mental health support and the percentage of the population that uses mental health services [54].

### Limitations

This study has some limitations. It focused on a single data set with 55 individuals and considered a specific type of feature (phone data based, as described by Singh and Long [8] in the past literature). The use of binary gender in the assessment is another limitation of this study. Although this study examined many of the commonly used ML methods, other approaches are well represented in the literature. Hence, we will be cautious in generalizing the results until they are supported at a scale with samples of more representative populations and many other ML algorithms. Future work may also suggest other bias reduction techniques to reduce the discriminatory outcomes of mental health assessment algorithms based on protected attributes. At the same time, this work is the first empirical effort to analyze the difference in the performance of mental health assessment algorithms based on gender. A key contribution of this study is the motivation for future work in this domain using varied data sets and methods.

### Conclusions

This study grounds the use of gender as a protected attribute to study fairness in phone-based mental health assessment algorithms. Mobile phones are now actively used by billions of individuals; hence, the automatic assessment of mental health using ML algorithms could potentially be beneficial in estimating and intervening in billions of individuals’ mental health conditions. An audit of commonly used ML algorithms for mental health assessment revealed that the performance of these algorithms can vary significantly depending on gender. This disparity in performance was found to be noticeably reduced after the application of a DIR approach by adapting the data used for modeling. The results move the literature forward on fairness in mental health assessment algorithms, particularly with gender as a protected attribute. Future work could consider larger data sets, protected attributes other than gender, and a newer approach to creating fair and accurate mental health assessment algorithms. Such results will pave the way for accurate and fair mental health support for all sections of society.

## Appendix

Multimedia Appendix 1 The 5 metrics to measure bias in machine learning algorithms.

## Abbreviations

∆FPR: delta false positive rate
∆TPR: delta true positive rate
BER: balanced error rate
DIR: disparate impact remover
IMEI: International Mobile Equipment Identity
ML: machine learning
RQ: research question
SAT: Scholastic Assessment Test
SPD: statistical parity difference
