# Supplementary material for: Fairness in Mobile Phone–Based Mental Health Assessment Algorithms: Exploratory Study
Source: JMIR Form Res. 2022 Jun 14;6(6):e34366. doi: 10.2196/34366 (PMC9240929; doi:10.2196/34366)
Supplement: Multimedia Appendix 1 [file formative_v6i6e34366_app1.docx]

Multimedia Appendix 1: [Five Metrics to measure bias in machine learning algorithms]

*Delta Accuracy*. It is one of the most intuitive and simple metrics used to quantify bias in the literature. The delta accuracy captures the difference in the accuracy of samples belonging to the privileged and unprivileged group where *S* is the protected (sometimes also called the *sensitive*) feature.

∆*accuracy* = *accuracy*(*S* = *privileged*) *− accuracy*(*S* = *unprivileged*) (1)

The definition of delta accuracy can be extended considering the difference in the true positive rate and the false positive rate.

*Delta True Positive Rate (*∆*TPR)*. The delta TPR metric focuses on the “equality of opportunity” aspect i.e., equal opportunity for truly *deserving* entries in both the privileged and the unprivileged groups to obtain a positive label (e.g., higher mental health label) from the algorithm [13,15].

∆*T P R* = *T P R*(*S* = *privileged*) *− T P R*(*S* = *unprivileged*) (2)

*Delta False Positive Rate (*∆*FPR).* The concept of “equalized odds'' extends the above idea of “equality of opportunity” to ensure that not only TPR but also FPR is equal across different groups [15,40]. The delta FPR refers to instances where undeserving candidates are granted positive outcomes.

∆*F P R* = *F P R*(*S* = *privileged*) *− F P R*(*S* = *unprivileged*) (3)

*Statistical Parity Difference (SPD)*. The SPD calculates the difference in the probability of favorable outcomes as assigned by the algorithm ($\hat{Y}=1$) being obtained by the unprivileged group compared to that of the privileged group [39]. For the model to be ideally fair, the statistical parity difference should be zero.

*SPD* = *P*($\hat{Y}$ = 1*|S* = *privileged*) *− P*($\hat{Y}$ = 1*|S* = *unprivileged*) (4)

*Disparate Impact*. Lastly, the disparate impact metric captures the ratio of the probability of favorable outcomes for the unprivileged group to that of the privileged group [16]. The ideal value for the disparate impact is supposed to be 1.0. For practical settings, however, a model is considered biased, if the value of the disparate impact is less than 0.8, indicating a preference or benefit for the privileged group [41]. If the disparate impact is greater than 1.0, then the model is considered to be biased against the privileged group [16].

$DI= \frac{P({\dot{\hat{Y}=1| S=unprivileged})}}{P({\dot{\hat{Y}=1| S=privileged})}}$ (5)
